# Pharmacokinetic modelling of *N*-(4-[^18^F]fluorobenzoyl)interleukin-2 binding to activated lymphocytes in an xenograft model of inflammation

**DOI:** 10.1007/s00259-012-2176-y

**Published:** 2012-07-10

**Authors:** Valentina Di Gialleonardo, Alberto Signore, Antoon T. M. Willemsen, Jurgen W. A. Sijbesma, Rudi A. J. O. Dierckx, Erik F. J. de Vries

**Affiliations:** 1 Department of Nuclear Medicine & Molecular Imaging, University Medical Center Groningen, Groningen, The Netherlands; 2 Nuclear Medicine Unit, Faculty of Medicine and Psychology, “Sapienza” University, Rome, Italy; 3 Medicina Nucleare, Ospedale S.Andrea, “Sapienza” University, Via di Grottarossa 1035, 00189 Rome, Italy

**Keywords:** Pharmacokinetics, [^18^F]FB-IL2, Molecular imaging, Inflammation

## Abstract

**Purpose:**

*N*-(4-[^18^F]Fluorobenzoyl)interleukin-2 ([^18^F]FB-IL2) specifically binds to interleukin-2 receptors (IL-2R) and thus may be used to detect inflammation processes using positron emission tomography (PET). We now validated whether [^18^F]FB-IL2 can be used to quantify activated human peripheral blood mononuclear cells (hPBMC) in rats by pharmacokinetic modelling.

**Methods:**

Eleven Wistar rats were subcutaneously inoculated in the shoulder with different amounts of phytohaemagglutinin (PHA) activated hPBMC 15 min before i.v. injection of [^18^F]FB-IL2. A 60-min dynamic PET scan was acquired and arterial blood sampling and metabolite analysis were performed. At the end of the scan, animals were terminated and the inflammatory lesion dissected. PET data were analysed using Logan and Patlak analysis as well as one-tissue and two-tissue compartment models. Model preferences according to the Akaike information criterion (AIC) and correlation between PET measurements and the number of CD25-positive cells were evaluated.

**Results:**

A high correlation between ex vivo tracer uptake (standardized uptake value) in the xenograft and the number of inoculated CD25-positive cells was observed (*R* ^2^ = 0.90). Plasma time-activity curves showed a rapid washout of the radiopharmaceutical from blood, while the time-activity curves of the inflammatory lesions showed slower washout. Time-activity curves could be fitted well by the Logan analysis method, indicating that the binding between [^18^F]FB-IL2 and CD25 is reversible. AIC indicated that data could be modelled best by a two-tissue reversible compartment model. A high correlation was observed between the binding potential and the number of CD25-positive cells (*R* ^2^ = 0.876, *p* < 0.0001). Based on binding potential measured by PET, the limit of detection was about 160,000 CD25-positive cells per 200 μl lesion (95 % confidence).

**Conclusion:**

[^18^F]FB-IL2 kinetics in this animal model of inflammation could be best described by a reversible two-tissue compartment model. The [^18^F]FB-IL2 binding potential is a suitable measure for accurate quantification of lymphocytic infiltration in pathological conditions with PET.

**Electronic supplementary material:**

The online version of this article (doi:10.1007/s00259-012-2176-y) contains supplementary material, which is available to authorized users.

## Introduction

High levels of interleukin-2 receptors (IL-2R) can be found mainly on the surface of activated T lymphocytes (both CD4+ and CD8+, mainly Th1 lymphocytes) [1–3] after endogenous stimulation. T lymphocytes are activated by inflammatory processes, such as tissue degeneration [4–6], autoimmune diseases [7, 8], viral, fungal and mycobacterial infections, graft rejection and in tumour infiltrates [9–15]. These disorders are characterized by the activation of the immune system and slow recruitment of immune cells (peripheral blood mononuclear cell, PBMC) in the lesion. The infiltration of PBMC in the inflammatory lesion is the histopathological hallmark of chronic inflammation, in contrast to the granulocyte infiltration found in acute inflammatory conditions. During activation, immune cells overexpress multimeric IL-2R, which are able to sustain immune cell proliferation and cell survival. The IL-2R consists of three subunits (α, β and γ chains, called CD25, CD122 and CD132, respectively). CD25 contains the main binding site for IL-2 and can be present as a transmembrane or soluble receptor. Because IL-2R expression is low in resting immune cells, the receptor might be a suitable biomarker to study active inflammation in chronic inflammatory diseases.

Since IL-2 is the natural ligand of the IL-2R, this glycoprotein was selected as a potential radiopharmaceutical for imaging activated T lymphocytes [16]. In the past, IL-2 was labelled with various isotopes for single proton emission computed tomography (SPECT) imaging [17–21]. The labelled IL-2 derivatives showed good results in terms of localization of the inflammation within the limitations of gamma camera imaging. Labelling with a suitable positron emission tomography (PET) isotope may improve sensitivity of this radiopharmaceutical and therefore *N*-(4-[^18^F]fluorobenzoyl)interleukin-2 ([^18^F]FB-IL2) was developed by our group. In our previous studies, we demonstrated the ability of [^18^F]FB-IL2 to selectively detect activated human peripheral blood mononuclear cells (hPBMC) in rodent models of inflammation by noninvasive PET imaging [22]. In addition, [^18^F]FB-IL2 was able to detect insulitis, a chronic inflammatory process that precedes the clinical onset of type 1 diabetes, in diabetes-prone rats and mice (data submitted for publication).

The detection of chronic inflammation is important for diagnostic, prognostic purposes and for monitoring the disease progression and the efficacy of treatment. To this end, quantitative measurement of the extent of lymphocytic infiltration is required. In the present study we aimed to investigate whether [^18^F]FB-IL2 PET is able to quantify the amount of activated lymphocytes in infiltrated tissue. For this purpose, pharmacokinetic modelling was performed. Wistar rats were subcutaneously inoculated with activated hPBMC and subsequently studied with [^18^F]FB-IL2 microPET. Dynamic PET data were analysed using various kinetics modelling approaches and correlated to the number of CD25-positive cells administered.

## Materials and methods

### Synthesis of [^18^F]FB-IL2

[^18^F]FB-IL2 was synthesized as described elsewhere [22]. [^18^F]FB-IL2 was obtained in 25 % radiochemical yield based on succinimidyl 4-[^18^F]fluorobenzoic acid ([^18^F]SFB) (corrected for the decay), with a specific activity of 117 ± 6 GBq/μmol at the end of synthesis and with a radiochemical purity >95 %.

### hPBMC activation and FACS analysis

Human blood was obtained from the local blood bank. hPBMC were isolated from human peripheral blood by density gradient medium centrifugation (Lymphoprep, Axis-Shield) using the rapid centrifugation procedure developed by Bøyum [23, 24]. Cells were kept in RPMI 1640 supplemented with L-glutamine, 10 % fetal calf serum (FCS), 100 IU penicillin/ml and 100 μg streptomycin/ml (all from GIBCO). Isolated hPBMC were incubated for 48 h with 5 μg/ml of phytohaemagglutinin (PHA-P, Sigma-Aldrich) at 37 °C and 5 % CO_2_ for cell activation. At the day of the microPET scan, overexpression of CD25 was determined by fluorescence-activated cell sorting (FACS) analysis. For this purpose, the cultured activated hPBMC were washed once with 3 ml of cold phosphate-buffered saline (PBS) and resuspended in PBS at a concentration of 10^6^ cells/ml. Aliquots (0.1 ml) of the cell suspension were incubated for 45 min in ice with PE-CD25 antibody or PE-IgG antibody (E-Bioscience) as controls. After being washed twice in 3 ml of cold PBS, the cells were resuspended in 0.5 ml of FACS solution (PBS supplemented with 5 % FCS) immediately prior to analysis using a FACSCalibur (BD Biosciences). FACS data were analysed using Winlist 5.0 software (Verity Software House) in order to determine the percentage of CD25-expressing cells in each sample.

### Animals

All animal experiments were carried out according to the Dutch Regulations for Animal Welfare. The protocol was approved by the Ethics Committee of the University of Groningen (protocol number: 5705 C).

After arrival, 11 Wistar rats (Charles River, The Netherlands) were acclimatized for at least 7 days. The animals were housed in Makrolon cages on a layer of wood shavings in a room with constant temperature (21 ± 2 °C) and 12-h light-dark regime. Commercial chow and tap water were available ad libitum. All rats were subcutaneously implanted with an increasing number of activated hPBMC in 100 μl PBS mixed with 100 μl of Matrigel (Becton Dickinson, The Netherlands). The activated cells were inoculated in the right shoulder of the animal 15 min before the PET experiment.

### PET acquisition protocol and arterial blood sampling

Rats were anaesthetized with 2 % isoflurane in medical air and a cannula for blood sampling was surgically inserted in the femoral artery. The animals were placed in a transaxial position in the PET camera (microPET Focus 220, Siemens/Concorde) with the shoulders of the rats in the field of view. Rats were injected trough the penile vein with 17.1 ± 1.2 MBq of [^18^F]FB-IL2 and a dynamic PET scan was immediately acquired for 60 min. After the emission scan, a transmission scan of 515 s with a ^57^Co point source was obtained for the correction of tissue attenuation. Two animals were scanned simultaneously in each scan session. Arterial blood was sampled during the course of the scan at 15, 30, 45, 60, 75, 90, 120, 180, 300, 450, 600, 900, 1,800 and 3,600 s after tracer injection. When a blood sample was collected, 0.1 ml of heparinized saline was injected via the artery cannula to prevent large changes in blood pressure. Blood samples were used to measure the plasma radioactivity concentration. Blood was centrifuged (5 min at 13,000 rpm) to separate cells from plasma. Then, 50 μl of each plasma sample was collected and the activity in these plasma samples was measured with a gamma counter (LKB Wallac, Turku, Finland). Plasma activity was corrected for decay.

### Radiometabolite analysis in rat plasma and urine

Radiometabolite analysis of rat plasma was performed in a separate study in rats without hPBMC lesion. Arterial blood samples were collected at 1, 10, 20, 30, 40 and 60 min after administration of 32.1 ± 9.8 MBq [^18^F]FB-IL2. Blood was centrifuged for 5 min at 13,000 rpm to separate the cells from the plasma fraction. After centrifugation, 5 μl of each plasma sample was spotted onto a thin-layer chromatography (TLC) plate (Merck F-254 silica gel strip). The strip was eluted at room temperature with ethyl acetate/*n*-hexane (3:1). [^18^F]SFB migrated with the solvent front (R_f_ = 1), and [^18^F]FB-IL2 remained at the origin (R_f_ = 0). The degradation product 4-[^18^F]fluorobenzoic acid ([^18^F]FBA) has an R_f_ = 0.7. Detection of the reference compounds on the TLC plates was performed by UV light (254 nm). For radiolabelled compounds, the detection on the TLC was performed by phosphor storage imaging (multisensitive screens, Packard). These screens were exposed to the TLC strips for a few minutes and subsequently read out using a Cyclone phosphor storage imager (PerkinElmer, USA) and analysed with OptiQuant software.

An additional 0.2 ml plasma sample obtained at 60 min was filtered through a Vivaspin filter with a 30 kDa cutoff; 0.1 ml of this filtrate was analysed by analytical HPLC for the identification of the radioactive metabolites. HPLC analyses were carried out with Elite LaChrom Merck Hitachi L-7100 pump system using Luna C18-column (5 μm, 4.6×250 mm) equipped with both UV (Elite LaChrom VWR Hitachi L-2400 UV detector set at 254 nm) and a Bicron radioactivity monitor. Gradient elution was performed using a mixture of 0.1 % aqueous trifluoroacetic acid (solvent A) and 0.1 % trifluoroacetic acid in acetonitrile (solvent B). The following gradient profile was used: 0–5 min 0 % B, 5–10 min 40 % B, 10–35 min 65 % B, 35–45 min 100 % B and 45–47 min 0 % B at flow rate of 1 ml/min. Retention times were: 24 min for [^18^F]FB-IL2, 26 min for [^18^F]FBA and 30 min for [^18^F]SFB. The same HPLC procedure was applied to analyse urine samples for the formation of radioactive metabolites, using samples collected 60 min after tracer injection.

### Ex vivo biodistribution

After the PET scan, Wistar rats were sacrificed by extirpation of the heart while under deep anaesthesia. The xenograft consisting of Matrigel mixed with inoculated cells was dissected and weighed. As a reference tissue muscle from the contralateral shoulder was isolated. Radioactivity in each sample was measured by an automated gamma counter (LKB Wallac, Turku, Finland). Radioactivity accumulation in the target lesion was expressed as standardized uptake value (SUV), using the formula: [(tissue activity concentration (MBq/g)]/[(injected dose (MBq)/body weight (g)].

### Image reconstruction

All emission scans were normalized and corrected for random coincidences, dead time, scatter, attenuation and decay. Emission sinograms were reconstructed using an ordered subset expectation maximization (OSEM) algorithm with 4 iterations and 16 subsets. Three-dimensional regions of interest (3-D ROIs) were generated automatically based on an intensity threshold method using Inveon Research Workplace software (Inveon, Siemens, USA). Briefly, all frames (0-60 min) were summed and an ROI of the inflammatory region was generated automatically with a 50 % threshold using a region growing method, i.e. only pixels were included with tracer uptake greater than 50 % of the maximum value within the lesion. The resulting ROIs were used on the original data set to create the corresponding time-activity curves (TACs) using standard software (Inveon, Siemens, USA).

### Kinetic analysis

Pharmacokinetic modelling of the tissue TACs was performed using standard software (Inveon, Siemens, USA). Graphical Logan analysis and Patlak analysis were used to determine the volume of distribution (V_d_). Two compartmental models were used to fit ROI data. These were a reversible one-tissue compartment model (1TCMR, one tissue compartment with two kinetic rate constants) and a reversible two-tissue compartment model (2TCMR, two tissue compartments with four kinetic rate constants). The blood pool compartment was considered as the input function using the present terminology [25]. Best-fit analysis was used to calculate the V_d_ and with the latter model also the binding potential (BP). V_d_ is defined as K1/k2 for the 1TCMR and (K1/k2)(1 + BP) for the 2TCMR, with the BP being equal to k_3_/k_4_. The optimal model was selected based on the Akaike information criterion (AIC) values generated by the analysis software. AIC selects the model with the best fit of the data, taking into account the number of fitted data points and the number of fitted parameters [26].

### Limit of detection

To assess the sensitivity of the PET method, the limit of detection (LOD) for CD25-positive cells was determined by measuring the BP of [^18^F]FB-IL2 in the contralateral shoulder (control). For this purpose, the ROI that was generated for the inflammatory lesion at the injection site was copied to the contralateral unaffected shoulder. The LOD was defined as the mean of the BP in the control tissue + 2 times the standard deviation in order to assure with 95 % confidence that a signal at the inflammatory lesion was not due to statistical variability. Subsequently, the LOD was applied to calculate the minimum number of CD25-positive cells that can be detected with [^18^F]FB-IL2, using the correlation between the number of CD25-positive cells and the BP (Fig. 7).

### Statistical analysis

All data are expressed as mean ± standard deviation. Correlations were calculated with the linear regression algorithm in Sigma Plot and were considered statistically significant whenever *R* ^2^ > 0.5 and *p* < 0.05.

## Results

### FACS analysis

The percentage of CD25-positive cells was calculated on the day of the microPET experiment. PHA-P caused variable stimulation of overexpression of CD25 on the cell surface of hPBMC. The fraction of activated cells ranged between 5.5 and 9.9 %. The number of the inoculated hPBMC was corrected for the fraction of CD25-positive cells before correlation with the PET results. The number of CD25-positive cells injected in the shoulder of the rats ranged from 0.17 × 10^6^ to 1.9 × 10^6^.

### Ex vivo measurement of tracer accumulation

**Fig. 1:**
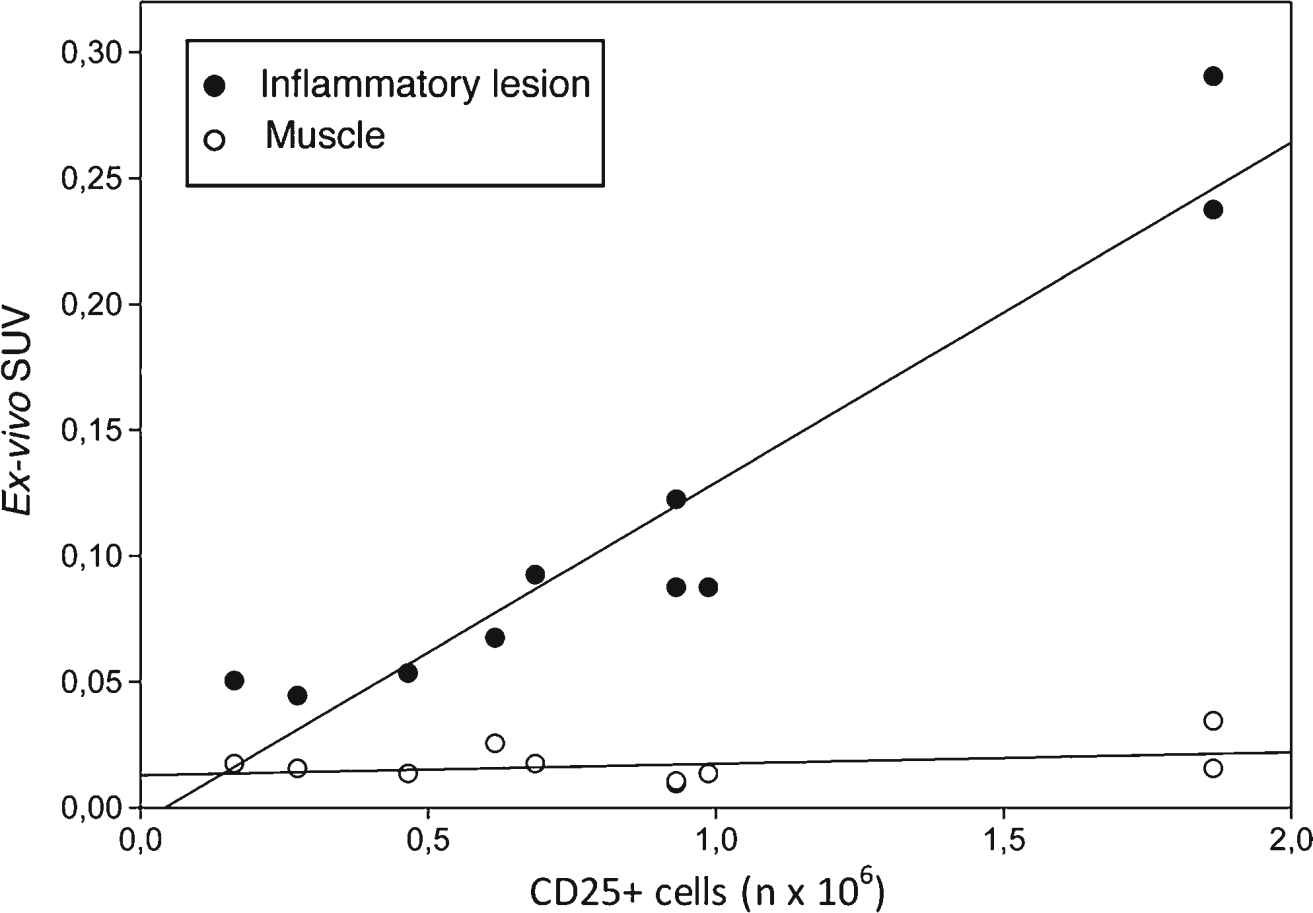
Correlation between [^18^F]FB-IL2 uptake (ex vivo SUV) and the number of CD25+ cells in the inflammatory lesion (*closed symbols*) and in muscle as a reference tissue (*open symbols*). *R* ^2^ = 0.90, *p* < 0.0001, y = 0.14x−0.006 and *R* ^2^ = 0.13, *p* = 0.31, y = 0.005x + 0.013 for the inflammatory lesion and muscle, respectively

For comparison with the PET studies, ex vivo measurement of radioactivity accumulated in the inflammatory lesion was performed. The radiopharmaceutical uptake of the dissected Matrigel + hPBMC was calculated in ten animals. In one animal, the ex vivo tracer accumulation could not be determined due to an experimental error during dissection and only kinetic modeling was performed in this animal. Muscle in the shoulder contralateral with respect to the site of hPBMC inoculation was used as a control tissue. As shown in Fig. 1, there is a strong correlation between [^18^F]FB-IL2 uptake and the number of inoculated activated CD25+ cells (*R* ^2^ = 0.90, *p* value <0.0001). As expected, no correlation was found between muscle uptake and number of inoculated cells (*R* ^2^ = 0.13, *p* value = 0.31).

### Small animal PET imaging

**Fig. 2:**
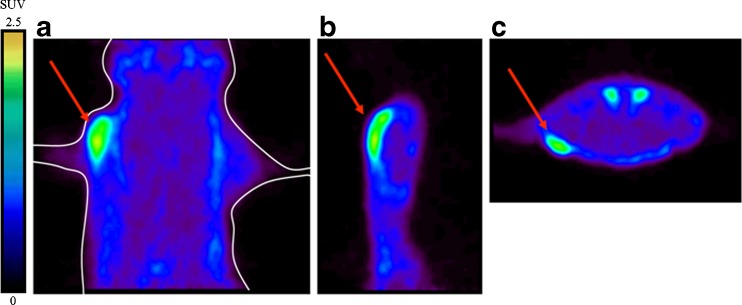
Small animal PET images of [^18^F]FB-IL2 in the inflammatory lesion of a rat inoculated with 0.99 × 10^6^ CD25+ cells. Coronal (**a**), sagittal (**b**) and transaxial (**c**) views of the thorax/abdomen of the rat. The inflammatory lesion is indicated by a *red arrow*. The image represents uptake in the inflammatory lesion from 0 to 60 min after injection of [^18^F]FB-IL2. In the transverse image, the submandibular glands are also visible

**Fig. 3:**
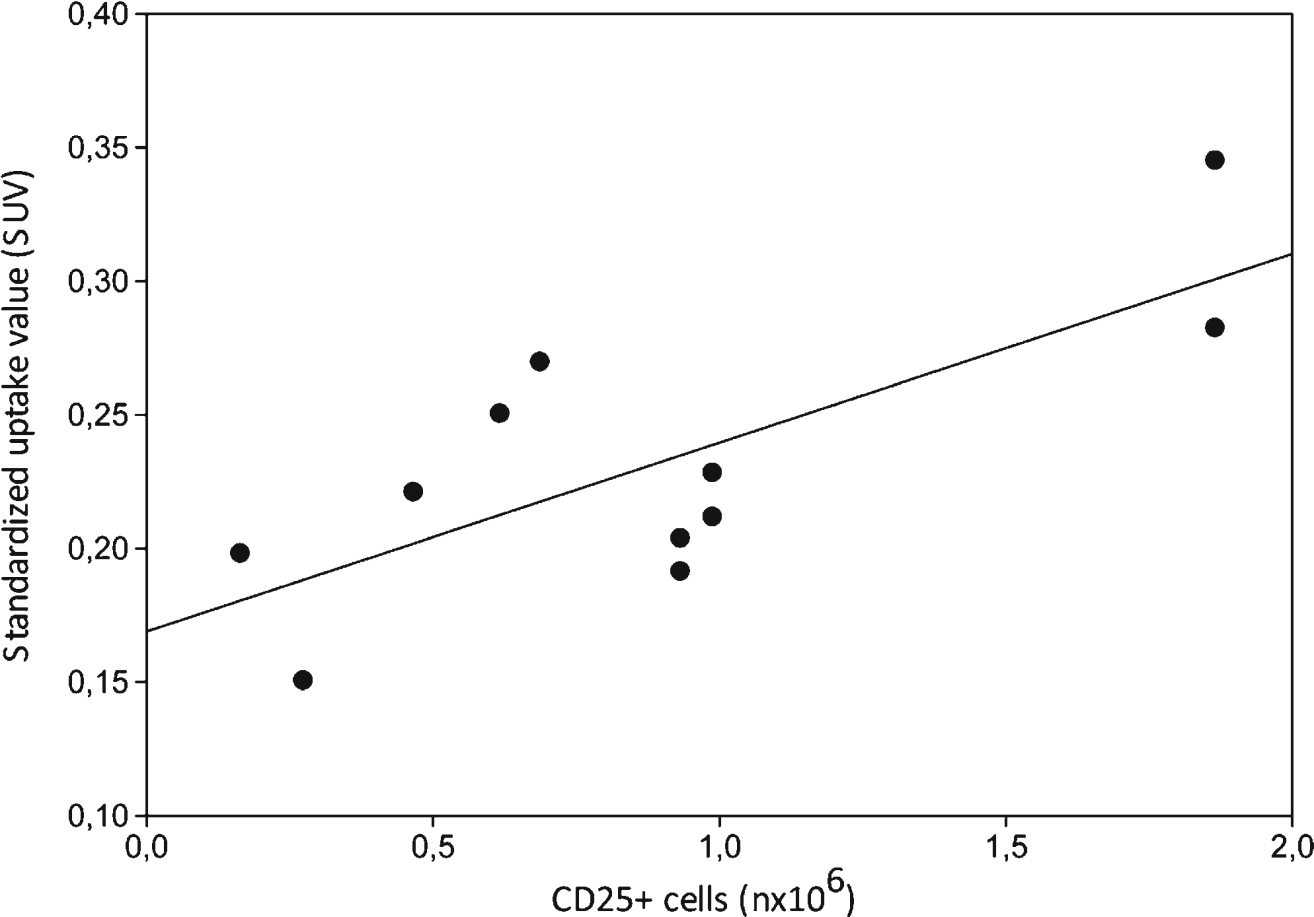
Correlation between PET imaging-derived [^18^F]FB-IL2 uptake expressed as SUV and the number of CD25+ cells present in the inflamed region. (*R* ^2^ = 0.56, *p* = 0.008, y = 0.07x + 0.17)

At 60 min after intravenous injection of [^18^F]FB-IL2, activated hPBMC could be clearly visualized in the right shoulder of all animals (Fig. 2). By applying 50 % of SUV_max_ as the threshold, an ROI was obtained for the inflammatory lesions. SUV values of [^18^F]FB-IL2 were calculated for each animal and were correlated with the number of CD25-positive cells in the inflamed region. As shown in Fig. 3, there was a moderate correlation between the cell number and the SUV of [^18^F]FB-IL2 PET (*R* ^2^ = 0.56, *p* = 0.008).

### [^18^F]FB-IL2 kinetics

**Fig. 4:**
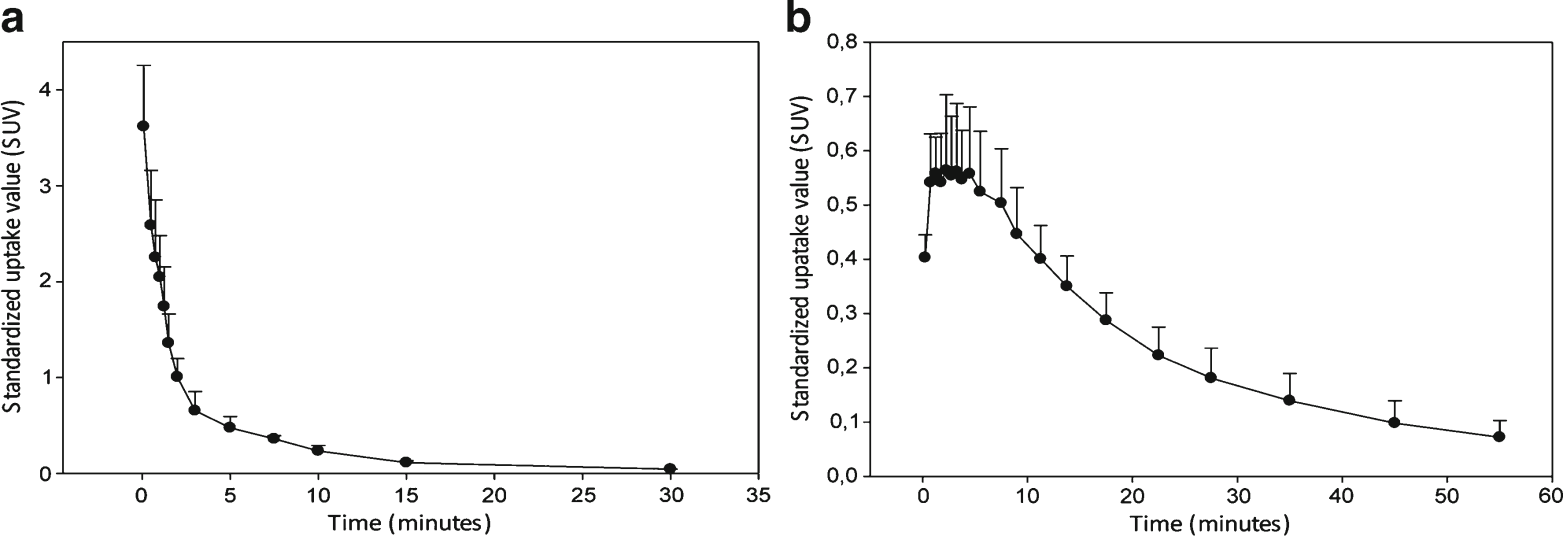
[^18^F]FB-IL2 time-activity curves **a** for plasma and **b** for the inflamed region (mean ± standard deviation of animals injected with 10^6^ CD25+ human activated PBMC)

Figure 4a shows the blood clearance of [^18^F]FB-IL2. Biexponential curve-fitting analysis of [^18^F]FB-IL2 blood clearance showed a two-phase blood clearance with 25 ± 11 % of the injected activity having a half-clearance time of 0.71 ± 0.29 min and 75 ± 11 % of the injected activity having a half-clearance time of 8.4 ± 2.6 min. Figure 4b shows the average TAC for the inflammatory lesion in the animals inoculated with approximately 10^6^ CD25-positive hPBMC. In contrast to plasma, the clearance from the cell inoculation site is slower and it can be described by a one-phase curve (0–60 min) with a half-clearance time of 37 ± 4 min.

### Metabolite analysis

Metabolites in plasma were analysed by TLC and by HPLC. TLC analysis did not show any formation of hydrophobic metabolites or formation of [^18^F]FBA (hydrolysis product). HPLC analysis confirmed that at 60 min post-injection (p.i.), the fraction of intact [^18^F]FB-IL2 in plasma was still 97.7 ± 1.2 %. Urine samples were analysed as well. After 60 min of tracer distribution, the main excretion product is an unknown hydrophilic degradation product of [^18^F]FB-IL2 with a retention time of 22 min. [^18^F]FBA and [^18^F]FB-IL2 (26 and 24 min, respectively) were not found in the urine. Because of the high stability of the tracer in plasma, the total activity in plasma without correction for metabolites was used as input function for the pharmacokinetic modelling study.

### Pharmacokinetic modelling

**Fig. 5:**
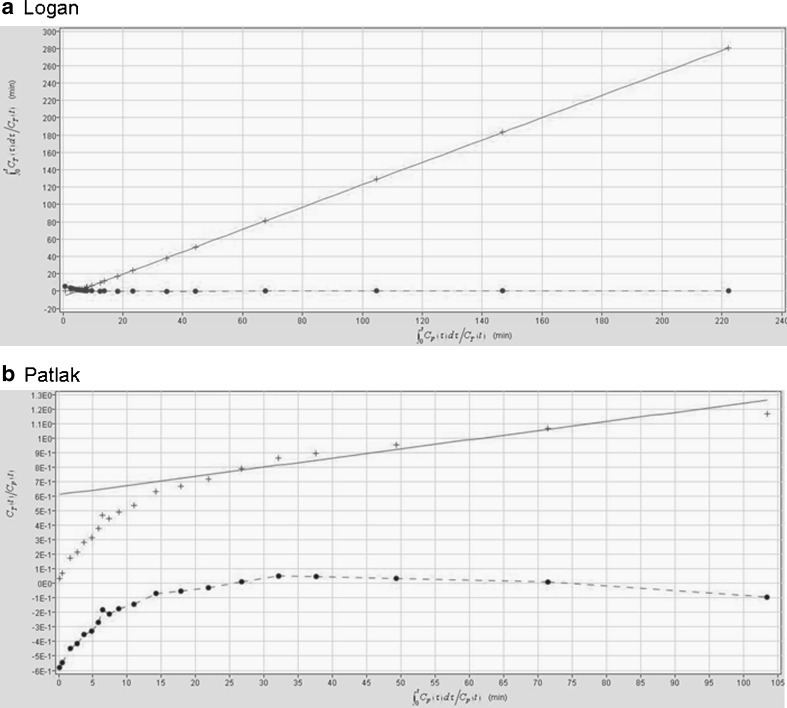
Representative Logan and Patlak graphical analysis for the inflamed region. **a** Logan graphical analysis showing a good fit of the inflamed region from 3 min after [^18^F]FB-IL2 injection, whereas **b** Patlak graphical analysis shows a delay time of 15 min

Pharmacokinetic modelling using the [^18^F]FB-IL2 TACs at the inoculation site from 0 to 60 min after tracer injection showed a significantly better fit (*p* < 0.0001) using Logan graphical analysis (*R* ^2^ = 0.97 ± 0.02) than with Patlak analysis (*R* ^2^ = 0.81 ± 0.12). In addition, Logan analysis required a delay time of only 3 min, whereas for Patlak analysis a delay time of 15 min had to be applied to obtain a reasonable fit (Fig. 5). This suggests that the binding between the fluorinated IL-2 and CD25 is better described by a reversible model.

**Fig. 6:**
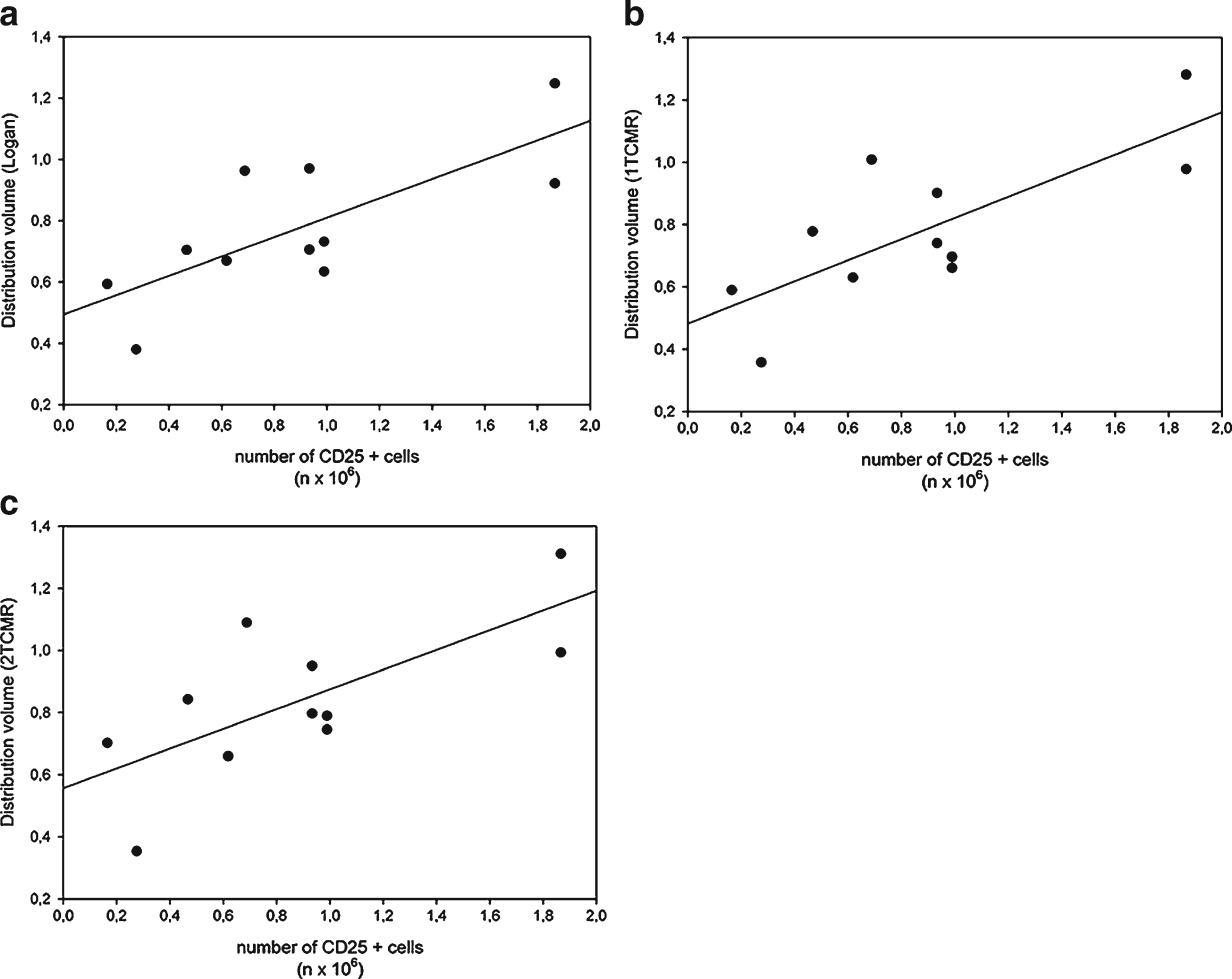
Correlation between the volume of distribution (V_d_) determined by **a** Logan analysis, **b** 1TCMR and **c** 2TCMR and the number of CD25-positive cells. *R* ^2^ was 0.59, *p* = 0.006, y = 0.32x + 0.49 for Logan analysis. *R* ^2^ was 0.59, *p* = 0.005, y = 0.34x + 0.48 for 1TCMR, and *R* ^2^ was 0.52, *p* = 0.013, y = 0.32 x + 0.56 for 2TCMR

Next, the distribution volume was calculated by Logan graphical analysis and compartment analysis using a 1TCMR and a 2TCMR model. Correlation between the number of CD25+ cells and the distribution volume are displayed in Fig. 6. The distribution volume showed a moderate correlation with the number of CD25+ cells when either Logan analysis or compartment analysis with a 1TCMR or 2TCMR model was used (*R* ^2^ = 0.59, 0.59 and 0.52, respectively).

**Fig. 7:**
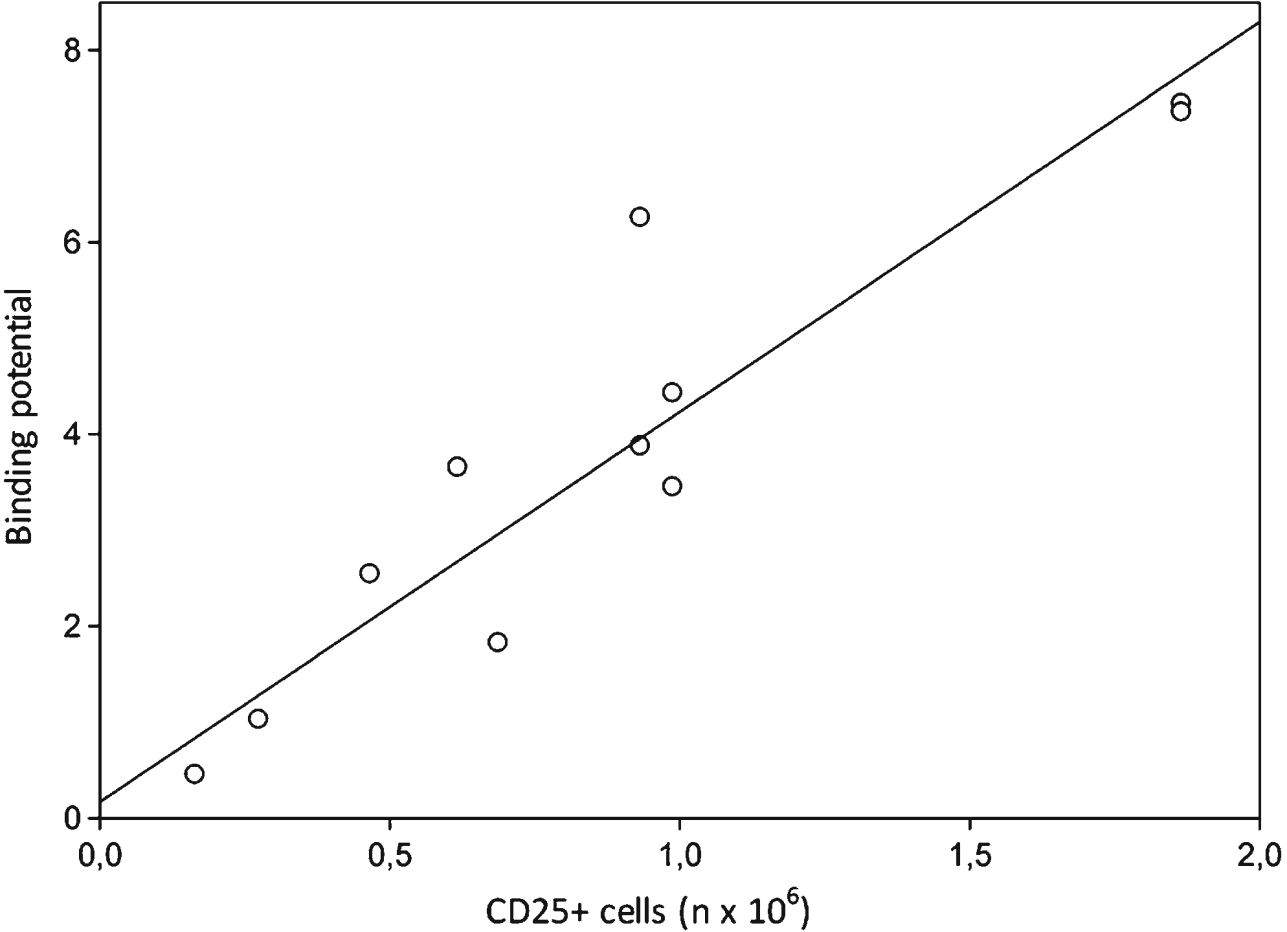
Correlation between the binding potential and the number of CD25-positive cells. *R* ^2^ 0.88, *p* < 0.0001, y = 4.06x + 0.17

The model of preference for compartment modelling was selected based on AIC values. The AIC values are 273 ± 16 and 241 ± 23 for 1TCMR and 2TCMR, respectively, with a highly significant difference between those models (*p* = 0.0011). Thus, TACs could be fit best using a 2TCMR model. In contrast to the distribution volume and the SUV, the BP is only dependent on the receptor binding characteristics of the radiopharmaceutical. To exclude any influence of differences in perfusion between animals, BP were calculated using the 2TCMR model. The number of CD25-positive cells and the corresponding BP values are plotted in Fig. 7. The BP is 0.45 for the lesion with 0.17 × 10^6^ CD25-positive cells and gradually increases up to 7.44 for the lesion with 1.9 × 10^6^ CD25+ cells. The BP increases by a factor of 4 for each million cells preset in the inflammatory lesion. There is a strong correlation between the BP and the number of CD25-positive cells (*R* ^2^ 0.88, *p* < 0.0001).

### Limit of detection

To assess the sensitivity of [^18^F]FB-IL2, the LOD was determined by measuring the background signal in the contralateral untreated shoulder. The average BP of [^18^F]FB-IL2 in the contralateral shoulder was 0.42 ± 0.20. Using a 95 % confidence interval, the LOD can be defined as the average background signal plus 2 standard deviations. In this model, the LOD of [^18^F]FB-IL2 would therefore be equal to 0.82. Using the equation for the correlation between the BP and the number of CD25-positive hPBMC (Fig. 7), we calculated that the lowest number of CD25-positive cells that can be reliably (95 % confidence) detected by this methods is approximately 160,000 cells in a lesion with a volume of 200 μl.

## Discussion

The present investigation aims to demonstrate that [^18^F]FB-IL2 PET imaging is a suitable tool to quantify lymphocyte infiltration in tissues. In this study, pharmacokinetic analysis of [^18^F]FB-IL2 was carried out in a rat xenograft model of inflammation. Different parametric methods were used to investigate which method was best to quantitatively measure activated immune cell infiltration.

Wistar rats inoculated with different numbers of human activated PBMC were selected as the animal model of choice. The big advantage of this animal model is the possibility to determine the exact number of inoculated CD25-positive target cells by measuring PHA-P-induced CD25 expression using FACS analysis. PHA-P is a strong activator of hPBMC. One of the most important markers of this activation process is the alpha subunit of the IL-2R (CD25). PHA-P-mediated cell activation depends on several factors such as time of incubation and amount of hPBMC in culture. Moreover, hPBMC derived from different healthy subjects show different predisposition to the activation. In this study, hPBMC were isolated from blood obtained from the local blood bank. hPBMC in these samples are less prone to activation by PHA-P than freshly isolated hPBMC from peripheral blood. Consequently, the fraction of CD25-expressing cells in the cell cultures was relatively low in this study, but the experimental design of this study allowed us to correct the data for the exact number of CD25-positive cells inoculated in the inflammatory lesion.

Ideally, an internal control could have been added to this model by injection of an equal number of non-activated hPBMCs or Matrigel alone in the contralateral shoulder of the animal. In a previous study in mice, however, we observed that inoculation of the Matrigel without cells already induced a strong migration of the activated hPBMC to the contralateral shoulder [22]. This cell migration caused a variable decrease of the activated PBMC in the inoculation site. Consequently, injection of a control xenograft would not allow us to have an exact estimation of the number of CD25-positive cells present in the inflammatory lesion and thus would hamper correlation of tracer accumulation with the number of CD25-positive cells.

Although the model used in this study does not represent all aspects of inflammation, it is an ideal model for the validation of the quantitative analysis of activated T lymphocytes using [^18^F]FB-IL2 PET. Other animal models of inflammation may better mimic disease processes, but do not allow exact quantification of the number of CD25-positive cells and can only give an indirect and inaccurate estimation of the number of cells present in the inflammatory lesion. Therefore, these more physiological animal models do not allow accurate correlation of the PET signal with the number of CD25 cells. A complicating factor in our xenograft animal model could be the involvement of host immune cells. To avoid interference of the host immune response, the interval between inoculation and PET imaging was therefore kept as short as possible.

Uptake of [^18^F]FB-IL2 in the region of inoculated activated hPBMC, measured ex vivo as SUV, increased with the number of inoculated CD25-positive cells and an excellent correlation between these parameters was found (*R* ^2^ = 0.90).

MicroPET images showed clear [^18^F]FB-IL2 uptake at the cell inoculation site, even for the smallest number of injected cells (165,000 cells). A moderate correlation was found between the number of CD25-positive cells and the SUV determined from PET data (*R* ^2^ = 0.56). This correlation obtained from the imaging study was substantially lower than the one from the ex vivo studies. This discrepancy might be explained by partial volume effects, which can underestimate the uptake in small or heterogeneous target regions. In this study, inflammatory lesions were relatively small (injection volume 0.2 ml), when compared to the spatial resolution of the PET camera (1.35 mm full-width at half-maximum). In addition, inflammatory lesions could be heterogeneous, because of the inhomogeneous presence of the Matrigel in the subcutaneous layer where the hPBMC are trapped. Furthermore, the inflammatory lesions are irregularly shaped. The inhomogeneity and irregular shape of the lesion could lead to differences in influence of the partial volume effect between lesions. Moreover, inflammation is a process that involves several changes in the inflammatory lesion, such as change in tissue perfusion and vessel permeability, which can reduce the usefulness of SUVs. Therefore, pharmacokinetic modelling might be required for accurate quantification of lymphocyte infiltration.

For the pharmacokinetic modelling, we used the total [^18^F]FB-IL2 plasma TAC without correction for tracer metabolites as the input function, since [^18^F]FB-IL2 was highly stable in plasma. More than 97 % of the tracer remained intact in plasma 60 min p.i. In contrast, only radioactive metabolites were found in urine. This suggests that [^18^F]FB-IL2 is metabolized by the kidneys and subsequently secreted into the urine. This observation is in agreement with the fate of intravenously injected native IL-2, which is also cleared into the urine after renal tubular catabolism [27].

Because the tracer is directly injected into the systemic circulation, the peak of the activity concentration in plasma of [^18^F]FB-IL2 reaches its maximum value within 15 s after tracer injection and then drops progressively. The plasma [^18^F]FB-IL2 concentration initially declines quite rapidly with an α phase of 0.71 ± 0.29 min due to the elimination from the plasma and the distribution of the tracer in tissue. The elimination of tracer from plasma is described by a β phase with a half-life of 8.4 ± 2.6 min. In contrast to the plasma curves, the TACs of the inflammatory lesion show a clearance half-life of 37 ± 4 %. These data suggest that after an initial nonspecific distribution of the tracer to the entire body of the animal [^18^F]FB-IL2 is retained in the inflammatory lesion because of the presence of target cells that overexpress the IL-2R.

Logan and Patlak analysis are simple graphical methods to estimate reversible and irreversible binding of a tracer to the target, respectively. We used these methods to study the binding characteristics of [^18^F]FB-IL2. The TACs of the hPBMC xenografts showed a better fit with Logan analysis than with the Patlak analysis method, indicating that binding between [^18^F]FB-IL2 and the IL-2R could be reversible. Compartment modelling showed that the kinetics of [^18^F]FB-IL2 are best described by a 2TCMR. However, the volume of distribution derived from Logan, 1TCMR or 2TCMR showed only moderate correlations with the cell number present in the inflammatory lesion. In contrast, BPs are strongly correlated with the number of CD25-positive cells present in the inflammatory lesion (*R* ^2^ = 0.88). The BP increases proportionally with the increase of the number of activated hPBMC in the inflammatory region, suggesting that with this method changes in the number of activated hPBMC should be easily detectable.

Despite the fact that we did not include a control lesion in the contralateral side, we were still able to estimate the amount of nonspecific uptake at the inflammatory lesion from the correlations of the tracer uptake with the number of CD25-positive cells. In fact, Figs. 3 and 6 show that both the curves of the SUV and the distribution volume as functions of the number of CD25-positive cells do not cross the y-axis at the origin, indicating that an SUV of approximately 0.17 and a distribution volume of 0.5 are due to nonspecific uptake (i.e. in case the number of CD25-positive cells is 0). However, when the tracer uptake is expressed as the BP the nonspecific binding is negligible, as the curve in Fig. 7 almost crosses the y-axis at the origin. This again stresses the importance of pharmacokinetic modelling to compensate for nonspecific effects on tracer uptake.

Taken together, our data clearly demonstrate that [^18^F]FB-IL2 BP is a better measure of activated hPBMC in the inflammatory lesion than the distribution volume or SUV. Both the distribution volume and the SUV depend not only on the binding characteristics of the tracer to IL-2R, but also on the perfusion of the tracer into the lesion. In contrast, BP is independent of tracer influx (K_1_) and efflux (k_2_), but only depends on the ratio of receptor binding and release.

Besides accuracy, sensitivity is also an important property of a diagnostic tool. Therefore, we determined the LOD of [^18^F]FB-IL2 PET and demonstrated that this technique can detect reliably as little as 160,000 CD25+ cells, if the radiopharmaceutical uptake is determined as BP. Moreover, this method is also sensitive with respect to detection of differences in CD25+ cell number. A difference in the number of CD25+ cells of 1 million leads to a difference in 4 BP units.

### Conclusion

In conclusion, the kinetics of [^18^F]FB-IL2 in an inflammatory lesion is well described by Logan graphical analysis and compartment modelling with a 2TCMR. Since [^18^F]FB-IL2 is stable in plasma, total plasma radioactivity without correction for metabolites can be used as the input function. Because of the high correlation between the BP and the number of CD25-positive cells in the inflammatory lesion, infiltration of activated hPBMC can be best quantified by measuring the BP with [^18^F]FB-IL2 PET. Quantification of the tracer uptake as SUV or distribution volume is less accurate, as these parameters are not only dependent on receptor binding characteristics, but also on perfusion effects. The results of this study indicate that [^18^F]FB-IL2 PET could provide hitherto unavailable opportunities to assess lymphocytic infiltration in inflammatory diseases in a non invasive and quantitative manner. The technique is likely sensitive enough to detect even low numbers of infiltrating lymphocytes or small changes in the inflammatory response.

## Electronic supplementary material

Below is the link to the electronic supplementary material.

ESM 1 (PDF 309 kb)
